# Dupilumab Can Induce Remission of Eosinophilic Gastritis and Duodenitis: A Retrospective Case Series

**DOI:** 10.14309/ctg.0000000000000646

**Published:** 2023-09-27

**Authors:** Twan Sia, Leeon Bacchus, Riki Tanaka, Raisa Khuda, Shibani Mallik, John Leung

**Affiliations:** 1 Boston Specialists, Boston, Massachusetts, USA;; 2 Stanford University, School of Medicine, Stanford, California, USA.

**Keywords:** eosinophilic gastrointestinal disorder, EGID, non–EoE-EGID, eosinophilic duodenitis, EoG

## Abstract

**INTRODUCTION:**

Noneosinophilic esophagitis eosinophilic gastrointestinal disorders (non–EoE-EGIDs) have limited treatment options to induce histologic and clinical remission. Dupilumab is a human monoclonal antibody against the interleukin-4 receptor ɑ subunit, which has been reported to induce improvement in pediatric patients with non–EoE-EGIDs.

**METHODS:**

We conducted a retrospective chart review to identify if patients with eosinophilic gastritis (EoG) and/or eosinophilic duodenitis (EoD) experience clinical and histologic remission with dupilumab.

**RESULTS:**

Twelve patients were included (2 patients with EoG and EoD, 3 patients with EoG only, and 7 patients with EoD only). All patients experienced improvement of at least 1 symptom on dupilumab, 3 patients (25%) had no change in severity of 1 or more of their symptoms, and no patients had worsening symptoms. On dupilumab, 2 patients with EoG (40%) and 3 patients with EoD (33.3%) were completely asymptomatic. Histologic changes were investigated in a subanalysis including 8 patients (2 patients with EoG and EoD, 2 patients with EoG only, and 4 patients with EoD only). Median peak gastric eosinophil counts in patients with EoG reduced from 80.5 eos/hpf (min–max 32–150, Q1–Q3 45.5–111) to 7.5 eos/hpf (min–max 0–28, Q1–Q3 1.5–16.8). Median peak duodenal eosinophil counts in patients with EoD reduced from 39 eos/hpf (min–max 30–50, Q1–Q3 37.3–46.3) to 16.5 eos/hpf (min–max 0–50, Q1–Q3 8–38.5). All 4 patients (100%) with EoG and 4 patients (66.6%) with EoD had histologic remission on dupilumab.

**DISCUSSION:**

In this retrospective case series, we showed preliminary evidence that dupilumab may be effective in inducing histologic and symptomatic remission in patients with non–EoE-EGIDs.

## INTRODUCTION

Eosinophilic gastrointestinal disorders (EGIDs) are a group of Th2-mediated disorders that are distinguished based on the location of eosinophilia in the gastrointestinal tract. Eosinophilic esophagitis (EoE) is the most well-studied EGID with standardized diagnostic criteria, extensive treatment algorithms, and well-characterized epidemiology and pathophysiology. By comparison, these are ill-defined in non–EoE-EGIDs such as eosinophilic gastritis (EoG) and eosinophilic duodenitis (EoD) (1). EoG and EoD are characterized by symptoms in the upper gastrointestinal tract and elevated eosinophils in the mucosa of the stomach and duodenum, respectively. Although there is a growing interest in EoG and EoD, they remain heavily under-researched (2–4). Prospective clinical trial data in EoG and EoD have been limited, and current treatment options are poor (5,6).

Dupilumab, an injectable human monoclonal antibody against the interleukin-4 receptor ɑ subunit, has been shown to be effective at inducing histoclinical remission in EoE (7). Given its safety and efficacy in EoE, dupilumab has been suggested to be a potential therapeutic in non–EoE-EGIDs (8). Currently, there is limited data supporting the use of dupilumab as a treatment for non–EoE-EGIDs (9,10). Therefore, in our study, we aim to describe the clinical and histologic effects of dupilumab in patients with EoG and/or EoD. Given the efficacy of dupilumab in treating EoE (7) and previous reports of dupilumab as a treatment in pediatric patients with non–EoE-EGIDs (9,10), we hypothesize that dupilumab may induce histologic and clinical remission in patients with EoG and/or EoD.

## METHODS

We performed a retrospective chart review to identify patients with EoG and EoD who had tried dupilumab. The electronic medical record at a single medical clinic was searched using the *International Classifications of Disease, 10th revision* code K52.81 eosinophilic gastritis or gastroenteritis between January 2017 and March 2023. Patients were excluded from our cohort based on the following criteria: (i) the patient did not have histologic confirmation of EoG or EoD with gastric or duodenal biopsies; or (ii) the patient did not start dupilumab. All patients had at least 5 biopsies taken at each of the following sites during esophagogastroduodenoscopy (EGD): gastric body, gastric antrum, and duodenal bulb. Histologically active EoG was defined as ≥ 30 eos/hpf in at least 5 hpfs in the body or antrum, and histologically active EoD was defined as ≥ 30 eos/hpf in at least 3 hpfs in the duodenum (8,9).

Data were extracted from the electronic medical record by trained investigators. During abstraction, investigators were blinded to the study hypotheses. Each piece of data was independently obtained by at least 2 investigators. A third investigator resolved any conflicting or ambiguous data. After data abstraction, all patient data were deidentified such that authors were unable to identify individual patients. Patient demographics (age and sex) and histoclinical information (symptoms, peak eosinophil counts, and treatment course) were recorded from the electronic medical record. Each symptom was reported as a binary absent or present and quantified using the following scoring system: 0 = absent, 1 = mild, 2 = moderate, and 3 = severe. While scoring, investigators were blinded as to whether the patient was at baseline or on dupilumab. Patient demographics, histoclinical characteristics at baseline, and changes in symptoms before and after dupilumab initiation were analyzed using descriptive statistics.

A subanalysis identifying histologic effects of dupilumab was performed. To be included in the subanalysis, the patient must have had repeat EGD with gastric and duodenal biopsies after being on dupilumab for at least 6 weeks. In our subanalysis, the primary end point was histologic remission of EoD and/or EoG, which was defined as < 30 eos/hpf in the 5 most densely populated hpfs for EoG and < 30 eos/hpf in the 3 most densely populated hpfs for EoD.

This study was deemed to be exempt from institutional review board approval by the Western Institutional Review Board (WIRB-Copernicus Group; WCG IRB). Therefore, the need for patient consent was waived.

## RESULTS

**Figure 1.**
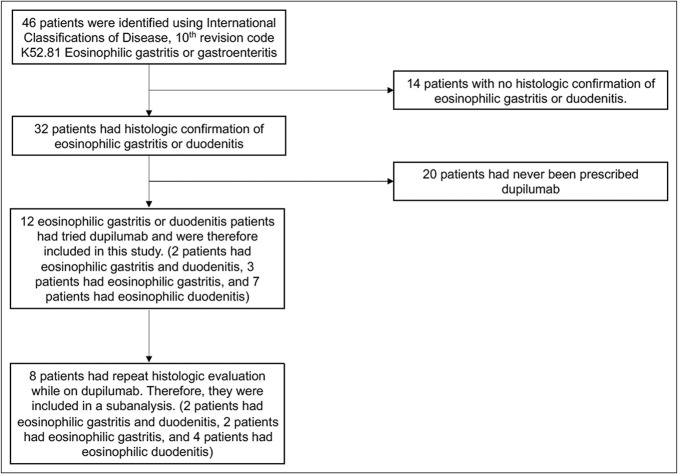
Flowchart of inclusion and exclusion criteria for included patients and for subanalysis.

A total of 46 patients were identified in our electronic medical record using the *International Classifications of Disease, 10th revision* code K52.81 eosinophilic gastritis or gastroenteritis. Of these patients, 14 had no histologic confirmation of EoG or EoD, and 20 had never been prescribed dupilumab. Therefore, 12 patients with histologically confirmed EoG and/or EoD who had tried dupilumab were included in this study (Figure 1).

Within our included patient cohort, 2 patients had both EoG and EoD, 3 patients had EoG without EoD, and 7 patients had EoD without EoG. The median age was 37.1 years (min–max 25.4–58.1, Q1–Q3 33.5–44.4), no patients were pediatric, and 7 patients were male (58.3%). Half of our patients (6 patients, 50%) had atopic comorbidities: allergic rhinitis (2 patients, 16.7%), asthma (1 patient, 8.3%), atopic dermatitis (4 patients, 33.3%), and food allergies (2 patients, 16.7%; Table 1). All 12 patients (100%) had EoE in addition to their non–EoE-EGID (Table 1). One patient (8.3%) had iron deficiency anemia. No other findings associated with EoG or EoD, such as protein-losing enteropathy, were identified in any of the other patients.

**Table 1.** Demographic information of all included patients and patients included for subanalysis of histologic effects of dupilumab

Characteristics	All included patients (n = 12)	Patients included for subanalysis (n = 8)
Male, n (%)	7 (58.3)	6 (75)
Age, median (min–max, Q1–Q3)	37.1 (25.4–58.1, 33.5–44.4)	35.5 (25.4–44.1, 31.9–39.5)
Pediatric, n (%)	0 (0)	0 (0)
Atopic comorbidity, n (%)	6 (50)	3 (37.5)
Allergic rhinitis	2 (16.7)	1 (12.5)
Asthma	1 (8.3)	0 (0)
Atopic dermatitis	4 (33.3)	3 (37.5)
Food allergies	2 (16.7)	1 (12.5)
Eosinophilic esophagitis comorbidity, n (%)	12 (100)	8 (100)
Eosinophilic gastritis and duodenitis, n (%)	2 (16.7)	2 (25)
Eosinophilic gastritis without duodenitis, n (%)	3 (25)	2 (25)
Eosinophilic duodenitis, without gastritis, n (%)	7 (58.3)	4 (50)

At baseline, all patients presented with symptoms. Patients with EoG presented with dysphagia (4 patients, 80%), abdominal pain (4 patients, 80%), bloating/gas (2 patients, 40%), diarrhea (3 patients, 60%), constipation (2 patients, 40%), nausea/vomiting (4 patients, 80%), and heartburn (5 patients, 100%). Upon EGD, patients with EoG had a median peak gastric eosinophil count of 111 eos/hpf (min–max 32–150, Q1–Q3 50–125). Patients with EoD also presented with dysphagia (8 patients, 88.9%), abdominal pain (8 patients, 88.9%), bloating/gas (2 patients, 22.2%), diarrhea (3 patients, 33.3%), constipation (3 patients, 33.3%), nausea/vomiting (6 patients, 66.7%), and heartburn (7 patients 77.8%). Upon EGD, patients with EoD had a median peak duodenal eosinophil count of 38 eos/hpf (min–max 30–139, Q1–Q3 36–45; Table 2).

**Table 2.** Histoclinical characteristics of all included patients with eosinophilic gastritis and or duodentitis

Characteristics	All included patients with eosinophilic gastritis at baseline (n = 5)	All included patients with eosinophilic gastritis on dupilumab (n = 5)	All included patients with eosinophilic duodenitis at baseline (n = 9)	All included patients with eosinophilic duodenitis on dupilumab (n = 9)
Peak gastric eos/hpf, median (min–max, Q1–Q3)	111 (32–150, 50–125)			
Peak duodenal eos/hpf, median (min–max, Q1–Q3)			38 (30–139, 36–45)	
Symptoms, n (%)				
Dysphagia	4 (80)	0 (0)	8 (88.9)	2 (22.2)
Abdominal pain	4 (80)	2 (40)	8 (88.9)	4 (44.4)
Bloating/gas	2 (40)	2 (40)	2 (22.2)	2 (22.2)
Diarrhea	3 (60)	3 (60)	3 (33.3)	2 (22.2)
Constipation	2 (40)	2 (40)	3 (33.3)	1 (11.1)
Nausea/vomiting	4 (80)	2 (40)	6 (66.7)	1 (11.1)
Heartburn	5 (100)	2 (40)	7 (77.8)	2 (22.2)
Asymptomatic	0 (0)	2 (40)	0 (0)	3 (33.3)

**Figure 2.**
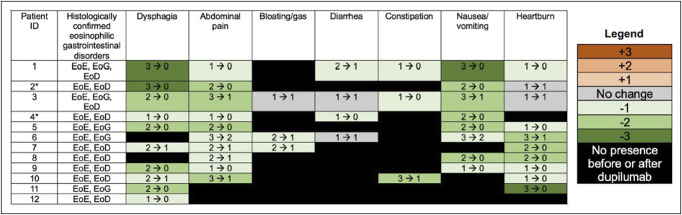
Summary plot of symptom scores before and after dupilumab initiation. Symptoms are reported as “baseline score → on dupilumab score.” Scores were given based on the following: 0 = absent, 1 = mild, 2 = moderate, 3 = severe. Shades of orange denote increase in score (worsening of symptoms), gray denotes no change in score, and shades of green denote decrease in score (improvement of symptoms) from baseline to on dupilumab. Black indicates that the symptom was absent before and after dupilumab initiation. Patients 1–8 are patients from the subanalysis for histologic effects of dupilumab. *Indicates patients who did not have histologic remission of EoD with dupilumab. EoE, eosinophilic esophagitis; EoD, eosinophilic duodenitis; EoG, eosinophilic gastritis.

Patients were started on 300 mg of dupilumab every week for their co-occurring EoE, followed by a repeat EGD for histologic evaluation. Dupilumab treatment course was a median of 36.9 weeks (min–max 6.1–54.9, Q1–Q3 15.9–50.4) for patients with EoG and 48.7 weeks (min–max 15.1–129.6, Q1–Q3 24.6–53) for patients with EoD. While on dupilumab, 2 patients (40%) with EoG and 3 patients (33.3%) with EoD had complete symptom remission (Table 2). To better characterize symptoms, scores were assigned to each symptom for each patient at baseline and on dupilumab. Changes in symptom scores from baseline to on dupilumab are summarized in Figure 2. No increase in symptom scores (worsening of symptoms) was noted in any of the 12 patients. Only 3 patients (25%) had a subset of symptoms without any change in scores. All other patients had a decrease in symptom scores. Of all 12 patients, only 1 patient reported mild joint pain, a previously reported adverse event associated with dupilumab (11). No other adverse events could be attributed to dupilumab.

A subanalysis was performed to identify whether dupilumab could induce histologic changes in patients with EoG and/or EoD. From the original 12 patients included in this study, 4 patients did not have a repeat EGD while on dupilumab. Thus, a total of 8 patients with histologically confirmed EoG and/or EoD who had a repeat histologic evaluation on dupilumab were included in this subanalysis (Figure 1).

Within the subanalysis cohort, 2 patients had EoG and EoD, 2 patients had EoG without EoD, and 4 patients had EoD without EoG (Figure 1). The median age was 35.5 years (min–max 25.4–44.1, Q1–Q3 31.9–39.5), no patients were pediatric, and 6 patients were male (75%). From the included 8 patients, 3 patients (37.5%) had atopic comorbidities: allergic rhinitis (1 patient, 12.5%), atopic dermatitis (3 patients, 37.5%), and food allergies (1 patient, 12.5%; Table 1).

**Figure 3.**
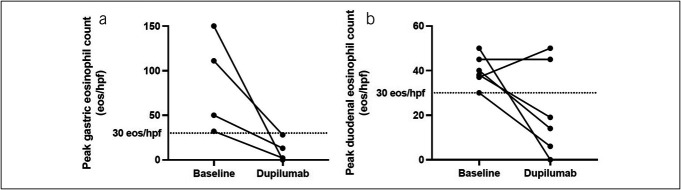
Histologic changes in patients included in subanalysis before and after dupilumab initiation. (**a**) Peak gastric eosinophil counts in patients with eosinophilic gastritis and (**b**) peak duodenal eosinophil counts in patients with eosinophilic duodenitis.

At baseline, patients with EoG had a median peak gastric eosinophil count of 80.5 eos/hpf (min–max 32–150, Q1–Q3 45.5–111), and patients with EoD had a median peak duodenal eosinophil count of 39 eos/hpf (min–max 30–50, Q1–Q3 37.3–46.3; Table 2). Patients were started on 300 mg of dupilumab every week. After a median of 30.7 weeks (min-max 15.1–54.9, Q1–Q3 21–45.8) of dupilumab for patients with EoG and 26.4 weeks (min–max 6.1–54.9, Q1–Q3 41.4) for patients with EoD, a repeat EGD was performed. The change in peak eosinophil counts for patients with EoG and EoD at baseline and on dupilumab are summarized in Figure 3. Median peak gastric eosinophil counts for all patients with EoG decreased to 7.5 eos/hpf (min–max 0–28, Q1–Q3 1.5–16.8). All 4 patients (100%) experienced histologic remission of EoG. Median peak duodenal eosinophil counts for all patients with EoD decreased to 16.5 eos/hpf (min–max 0–50, Q1–Q3 8–38.5), with 4 patients (66.7%) experiencing histologic remission of EoD (Table 2). All patients also experienced histologic remission of EoE while on dupilumab. Within this subanalysis cohort, all patients experienced some improvement in symptoms, including the 2 patients with EoD who did not have histologic remission with dupilumab (Figure 2).

**Figure 4.**
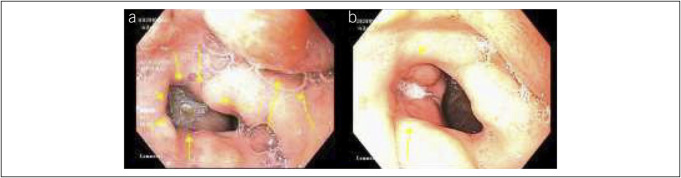
Endoscopic view of (**a**) a 30-mm antral ulcer in patient 6 at baseline and (**b**) a 10-mm antral ulcer in patient 6 after dupilumab initiation.

Of note, 1 patient (patient 6) with EoE and EoG had a 30-mm antral ulcer upon EGD at baseline. After treatment with dupilumab, a repeat EGD showed that the antral ulcer had decreased in size to 10 mm in diameter (Figure 4). Another patient (patient 1) had EoE, EoG, and EoD that was histoclinically unresponsive to topical corticosteroid, extensive food elimination diet, proton pump inhibitor, lirentelimab (anti–Siglec-8) therapy, and various combination therapies. At baseline and on other therapies, he was reliant on a percutaneous endoscopic gastrostomy tube for 14 years. After starting dupilumab, the patient noted symptom benefit, and the percutaneous endoscopic gastrostomy tube was able to be removed. The resulting fistula was closed using 3 hemostatic clips on the gastric side, cauterized with silver nitrate, and closed with tissue-adhesive glue on the cutaneous side. The patient has since been able to reintroduce foods.

## DISCUSSION

Previous literature regarding the use of dupilumab as a treatment for non–EoE-EGIDs is limited. A recent case series by Patel et al reported 2 pediatric patients with EGID refractory to standard treatment (patient 1 with EoE, EoG, and EoD; and patient 2 with EoE, EoG, EoD, and eosinophilic jejunitis [EoJ]) who were treated with dupilumab with histoclinical remission except for persistent EoJ in patient 2. Patel et al (9) also reported a third patient with EoE and EoJ in which histologic remission was induced with swallowed steroids and maintained when the patient was switched to dupilumab monotherapy. Another case report described a pediatric patient with EoG, EoD, and eosinophilic colitis who had clinical benefit with dupilumab, but histology was unavailable (10). Though novel, these case reports describe dupilumab improving symptoms in pediatric patients with EGID, but have incomplete histologic data, a key feature for identifying remission of EGID. In addition, these studies only report cases where dupilumab was successful in inducing histologic or clinical remission of EGID, therefore limiting our understanding of dupilumab's effectiveness.

Our study provides preliminary evidence that dupilumab may be a suitable therapy for inducing histologic and clinical remission of EoG and/or EoD. In our study of 12 patients with EoG and/or EoD, all patients experienced improvement of their symptoms, no patients experienced any worsening of symptoms, and only 3 patients (25%) experienced no change in 1 or more of their symptoms (Figure 2). On dupilumab, 2 patients with EoG (40%) and 3 patients with EoD (33.3%) were completely asymptomatic (Table 2). Our subanalysis of 8 patients with repeat histologic evaluation on dupilumab showed that median peak gastric eosinophil counts reduced from 80.5 eos/hpf (min–max 32–150, Q1–Q3 45.5–111) at baseline to 7.5 eos/hpf (min–max 0–28, Q1–Q3 1.5–16.8) while on dupilumab for patients with EoG and that median peak duodenal eosinophil counts reduced from 39 eos/hpf (min–max 30–50, Q1–Q3 37.3–46.3) at baseline to 16.5 eos/hpf (min-max 0–50, Q1–Q3 8–38.5) on dupilumab for patients with EoD (Figure 3, Table 3). On dupilumab, all 4 patients (100%) had histologic remission of EoG, and 4 patients (66.7%) had histologic remission of EoD (Table 3). To our knowledge, our study is the first to describe dupilumab usage in adult patients with EoG and/or EoD.

**Table 3.** Histoclinical characteristic of subanalysis patients with eosinophilic gastritis and/or duodenitis who had repeat histologic evaluation at baseline and on dupilumab

Characteristics	Eosinophilic gastritis subanalysis patients at baseline (n = 4)	Eosinophilic gastritis subanalysis patients on dupilumab (n = 4)	Eosinophilic duodenitis subanalysis patients at baseline (n = 6)	Eosinophilic duodenitis subanalysis patients on dupilumab (n = 6)
Peak gastric eos/hpf, median (min–max, Q1–Q3)	80.5 (32–150, 45.5–111)	7.5 (0–28, 1.5–16.8)		
Peak duodenal eos/hpf, median (min–max, Q1–Q3)			39 (30–50, 37.3–46.3)	16.5 (0–50, 8–38.5)
Histologic remission on dupilumab, n (%)		4 (100)		4 (66.7)
Symptoms, n (%)				
Dysphagia	3 (75)	0 (0)	5 (83.3)	1 (16.7)
Abdominal pain	4 (100)	2 (50)	6 (100)	3 (50)
Bloating/gas	2 (50)	2 (50)	2 (33.3)	2 (33.3)
Diarrhea	3 (75)	3 (75)	3 (50)	2 (33.3)
Constipation	2 (50)	0 (0)	2 (33.3)	0 (0)
Nausea/vomiting	4 (100)	2 (50)	5 (83.3)	1 (16.7)
Heartburn	4 (100)	2 (50)	5 (83.3)	2 (33.3)
Asymptomatic	0 (0)	1 (25)	0 (0)	1 (16.7)

Of interest, the 2 patients with EoD who did not experience histologic remission (peak duodenal eosinophil count was 45 eos/hpf at baseline to 45 eos/hpf on dupilumab, and 37 eos/hpf at baseline to 50 eos/hpf on dupilumab) had improvements in symptoms. Of these 2 patients, 1 experienced complete clinical remission, and the other was asymptomatic except for residual heartburn. This result suggests that clinical symptoms may not correlate with histologic findings in EoD, which is supported by previous studies noting a similar dissociation in EoE (12).

Of note, all 12 patients in our cohort had concurrent EoE, which was in histologic remission while on dupilumab. Therefore, dupilumab may be a good option for patients with EGID involving multiple parts of the gastrointestinal tract. However, it is also possible that dupilumab may only induce histologic remission of EoG and/or EoD in patients with concurrent EoE.

There are currently 2 clinical trials investigating the safety and efficacy of dupilumab in patients with EoG and/or EoD registered on ClinicalTrials.gov (NCT03678545, NCT05831176). Our preliminary results suggest that these trials may find favorable results supporting that dupilumab is safe and effective for inducing remission in patients with EoG and/or EoD.

Limitations include the completeness of the medical record, small sample size, and a single site, retrospective design, which precluded us from performing a more complete analysis. For example, we were also unable to reliably abstract endoscopic or other histologic features other than mucosal eosinophilia (such as degranulated or intraepithelial eosinophils, eosinophil glands or crypt abscesses, epithelial degeneration or regeneration, foveolar or crypt hyperplasia, small bowel villous atrophy, and other previously noted changes in EoG/EoD (13)) in a systematic manner. In addition, previous work studying the natural history of non–EoE-EGIDs found spontaneous remission in approximately 40% of cases (14). Therefore, we cannot preclude the possibility that a subset of our patients experienced spontaneous remission of their EoG and/or EoD, rather than remission being induced by dupilumab. Future prospective work investigating the efficacy of dupilumab in treating the histoclinical characteristics of non–EoE-EGIDs should be compared against a placebo group to account for the possibility of spontaneous remission.

## CONFLICTS OF INTEREST

**Guarantor of the article:** John Leung, MD.

**Specific author contributions:** All authors approved of the submitted manuscript. T.S.: conceptualization, data curation, formal analysis, investigation, methodology, validation, writing original draft, review and editing. L.B.: data curation, validation, writing original draft, review and editing. R.T.: data curation, validation, review and editing. R.K.: data curation, validation, review and editing. S.M.: data curation, validation, review and editing. J.L.: conceptualization, project administration, supervision, review and editing.

**Financial support:** This research received no specific grant from any funding agency in the public, commercial, or not-for-profit sectors.

**Potential competing interests:** J.L.: is a consultant for Devine; Millimet & Branch Professional Education; Sanofi; Huron Consulting Services LLC; Takeda; Ribon Therapeutics; Tegus; Slingshot; Guidepoint; Cowen; Regeneron; and AstraZeneca. None of the other authors have relevant conflicts of interests to disclose.

**Data sharing statement:** All relevant deidentified data and study materials are stored in an HIPPA-compliant, password-protected, cloud-based storage. Access to these files will be provided on reasonable request to the corresponding author, John Leung.

**Study Highlights**

**WHAT IS KNOWN**

✓ Current treatment options for noneosinophilic esophagitis eosinophilic gastrointestinal disorders (non-EoE-EGIDs) are limited.
✓ Previously, dupilumab was shown to improve non-EoE-EGIDs.

**WHAT IS NEW HERE**

✓ Dupilumab improved symptoms in all patients with eosinophilic gastritis and/or eosinophilic duodenitis, and induced histologic remission in a large proportion of patients.
✓ Dupilumab was effective in patients refractory to other treatment options, including lirentelimab.
